# Peripheral Blood Genes Crosstalk between COVID-19 and Sepsis

**DOI:** 10.3390/ijms24032591

**Published:** 2023-01-30

**Authors:** Changyin Fang, Yongping Ma

**Affiliations:** Department of Biochemistry and Molecular Biology, Basic Medical College, Chongqing Medical University, Yuzhong District, Yi XueYuan Road, No 1, Chongqing 400016, China

**Keywords:** COVID-19, sepsis, differentially expressed genes, protein–protein interaction, drug molecule

## Abstract

Severe coronavirus disease 2019 (COVID-19) has led to a rapid increase in death rates all over the world. Sepsis is a life-threatening disease associated with a dysregulated host immune response. It has been shown that COVID-19 shares many similarities with sepsis in many aspects. However, the molecular mechanisms underlying sepsis and COVID-19 are not well understood. The aim of this study was to identify common transcriptional signatures, regulators, and pathways between COVID-19 and sepsis, which may provide a new direction for the treatment of COVID-19 and sepsis. First, COVID-19 blood gene expression profile (GSE179850) data and sepsis blood expression profile (GSE134347) data were obtained from GEO. Then, we intersected the differentially expressed genes (DEG) from these two datasets to obtain common DEGs. Finally, the common DEGs were used for functional enrichment analysis, transcription factor and miRNA prediction, pathway analysis, and candidate drug analysis. A total of 307 common DEGs were identified between the sepsis and COVID-19 datasets. Protein–protein interactions (PPIs) were constructed using the STRING database. Subsequently, hub genes were identified based on PPI networks. In addition, we performed GO functional analysis and KEGG pathway analysis of common DEGs, and found a common association between sepsis and COVID-19. Finally, we identified transcription factor–gene interaction, DEGs-miRNA co-regulatory networks, and protein–drug interaction, respectively. Through ROC analysis, we identified 10 central hub genes as potential biomarkers. In this study, we identified SARS-CoV-2 infection as a high risk factor for sepsis. Our study may provide a potential therapeutic direction for the treatment of COVID-19 patients suffering from sepsis.

## 1. Introduction

Coronavirus disease 2019 (COVID-19) caused by severe acute respiratory syndrome coronavirus 2 (SARS-CoV-2) strains has become a pandemic worldwide [1,2]. The SARS-CoV-2 virus spreads rapidly through respiratory droplets and aerosols [3]. The most common symptoms of mild COVID-19 are fever, dry cough, sore throat, fatigue, and diarrhea [4,5,6]. Pathological manifestations of severe COVID-19 include pulmonary and multiple organ failure, immune activation, and uncontrolled cytokine responses [7,8]. Importantly, most COVID-19 patients eventually develop symptoms of septic shock, including cold limbs, microcirculation problems, and cytokine storms [9].

Sepsis is a systemic inflammatory response syndrome caused by the invasion of pathogenic microorganisms such as bacteria into the body [10]. Sepsis is life-threatening organ dysfunction caused by a dysregulated host response to infection [11]. Clinical manifestations of sepsis begin with inflammation and progress to circulatory organ dysfunction closely related to the production of pro- and anti-inflammatory cytokines, ultimately leading to multiple organ failure syndrome [11,12,13].

Although it presents important differences in pathogenesis, COVID-19 shares many similarities with sepsis in many aspects. It has been well established that both diseases involve cytokine storms, procoagulant states, toll-like receptor (TLR) signaling, pathogen-associated molecular patterns (PAMPs), and damage-associated molecular patterns (DAMPs) [14,15,16]. Furthermore, manifestations including increased heart rate, respiratory failure, fever, leukopenia, hypotension, leukocytosis, multiorgan dysfunction syndrome, coagulopathy, and septic shock are common to COVID-19 and sepsis [17,18,19,20]. In addition, as part of clinical care, the COVID-19 treatment guidelines, “surviving sepsis campaign”, are being implemented for seriously ill patients [21]. Given the similarities between COVID-19 and sepsis, it is necessary to determine the biological links and potential molecular mechanisms that link the two diseases, which may provide new insights into the pathogenesis of COVID-19 and sepsis and offer potential therapeutic agents for sepsis patients with COVID-19.

In this study, we identify common differentially expressed genes by performing differential analysis of blood RNA sequencing data from COVID-19 patients and sepsis patients, respectively. Bioinformatics systems biology approaches were successfully used to analyze the underlying molecular mechanisms and identify some drugs that may be useful for the treatment of COVID-19 and sepsis.

## 2. Result

### 2.1. The Identification of DEGs and Common DEGs between Sepsis and COVID-19

**Figure 1:**
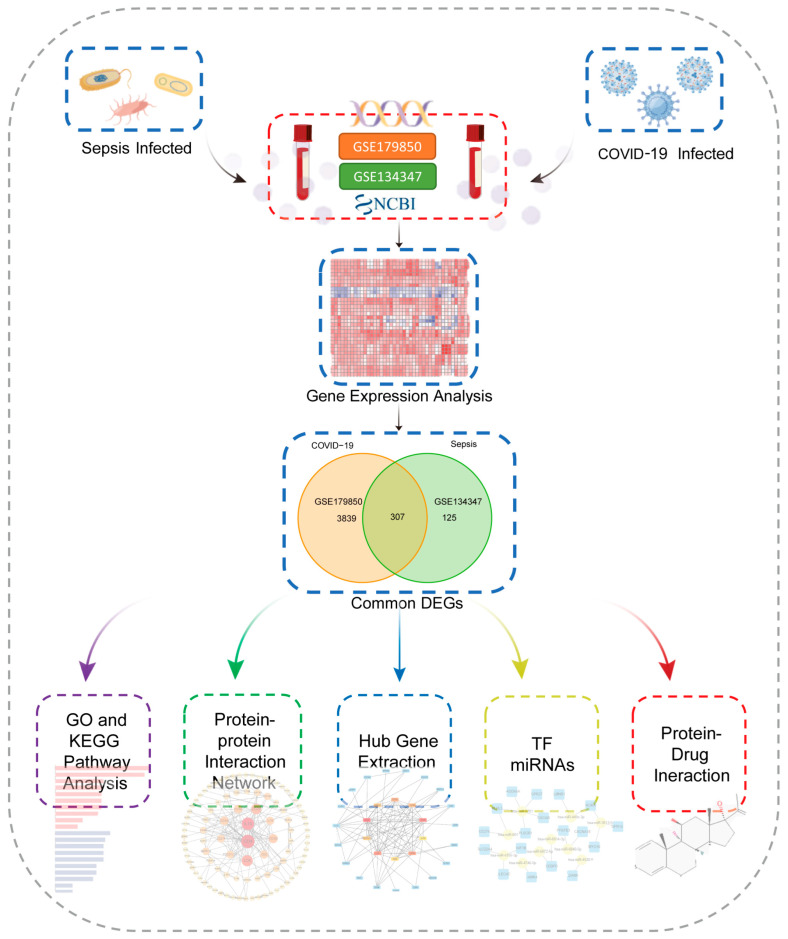
This is a diagram of the entire study workflow.

**Figure 2:**
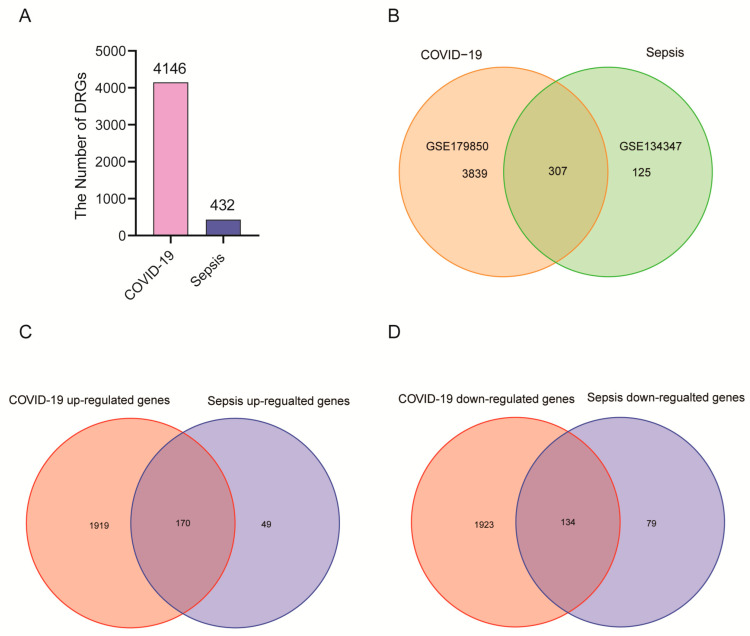
There are two GEO datasets included in the experiment: COVID-19 (GSE179850) and sepsis (GSE134347). (**A**) In a histogram, the number of genes that are differentially expressed between COVID-19 patients and sepsis patients. (**B**) There is an overlap between COVID-19 and sepsis differentially expressed genes. (**C**) The overlapping up-regulated genes between COVID-19 and sepsis. (**D**) The overlapping down-regulated genes between COVID-19 and sepsis.

This flowchart illustrates all the key steps in this study (Figure 1). We downloaded whole blood expression profiles from GEO and identified common differentially expressed genes for COVID-19 and sepsis to investigate the interrelationship and impact between sepsis and COVID-19. First, 4146 DEGs were screened in COVID-19, including 2089 up-regulated genes and 2057 down-regulated genes (Supplementary Table S1). Then, 432 DGEs were screened in sepsis, including 219 up-regulated genes and 213 down-regulated genes (Supplementary Table S2). Following the intersection of differentially expressed genes from the two datasets, 307 common DEGs were obtained (Figure 2A,B; Supplementary Table S3). Among them, 170 genes are up-regulated in both diseases and 134 genes are down-regulated (Figure 2C,D). These results indicated these genes share a high consistency in the direction of changes, and the underlying mechanisms of sepsis and COVID-19 may partially associate with each other.

### 2.2. GO and KEGG Enrichment Analysis

**Figure 3:**
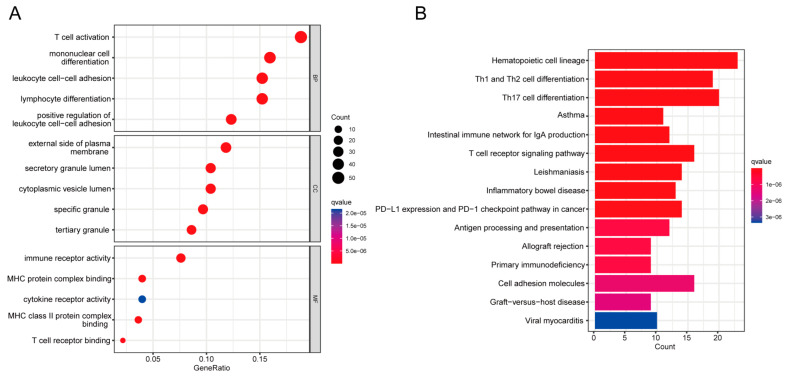
COVID-19 and sepsis genes with common differential expression were analyzed using GO and KEGG enrichment. (**A**) GO enrichment analysis histogram; (BP) biological process, (CC) cellular components, (MF) molecular function. (**B**) Bar plot of KEGG pathway analysis.

GO and pathway enrichment analyses were performed using the R “clusterProfiler” package to explore the biological features and enriched pathways of common DEGs. The GO database was used as the annotation source to perform GO analysis of three aspects: biological process (BP), cellular composition (CC), and molecular function (MF). We present the top five items for each category of GO terms in a bar chart. DEGs are significantly enriched in the regulation of T cell activation as well as mononuclear cell differentiation in biological process (BP) subsets. External side of plasma membrane and secretory granule lumen pathways are enriched in cellular compartment (CC) subsets. Pathways involved in immune receptor activity, MHC protein complex binding, and cytokine receptor activity are enriched in molecular function (MF) subsets (Figure 3A). KEGG is an online database for the systematic analysis of gene function, and reveals the interplay between genes and biological processes [22]. To explore the biological functions and enriched pathways of common DEGs, KEGG enrichment analysis was performed. The top fifteen pathways identified by KEGG pathway analysis are as follows: hematopoietic cell lineage, Th1 and Th2 cell differentiation, Th17 cell differentiation, asthma, intestinal immune network for IgA production, T-cell receptor signaling pathway, leishmaniasis, inflammatory bowel disease, PD-L1 expression and PD-1 checkpoint pathway in cancer, antigen processing and presentation, allograft rejection, primary immunodeficiency, cell adhesion molecules, graft-versus-host disease, and viral myocarditis (Figure 3B). The complete results of GO and KEGG enrichment analysis are shown in Supplementary Tables S4 and S5.

### 2.3. Identification of Hub Genes

**Figure 4:**
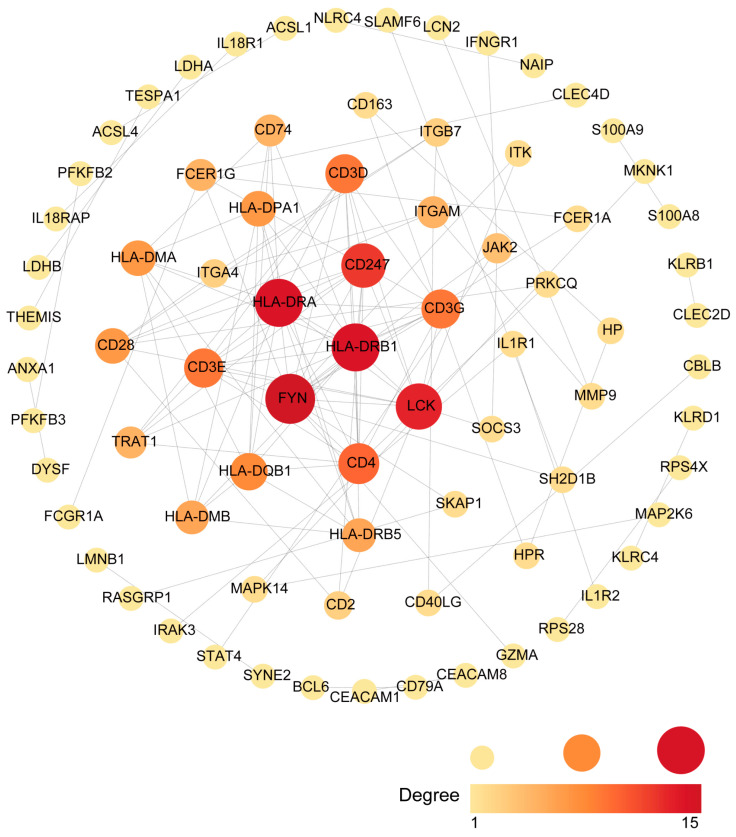
An analysis of the DEGs shared between COVID-19 and sepsis based on the PPI network. Cytoscape was used to visualize the PPI network generated by String. The darker the color, the more connections to the gene are identified. The larger size of the node suggests the higher connection degree of the gene.

**Figure 5:**
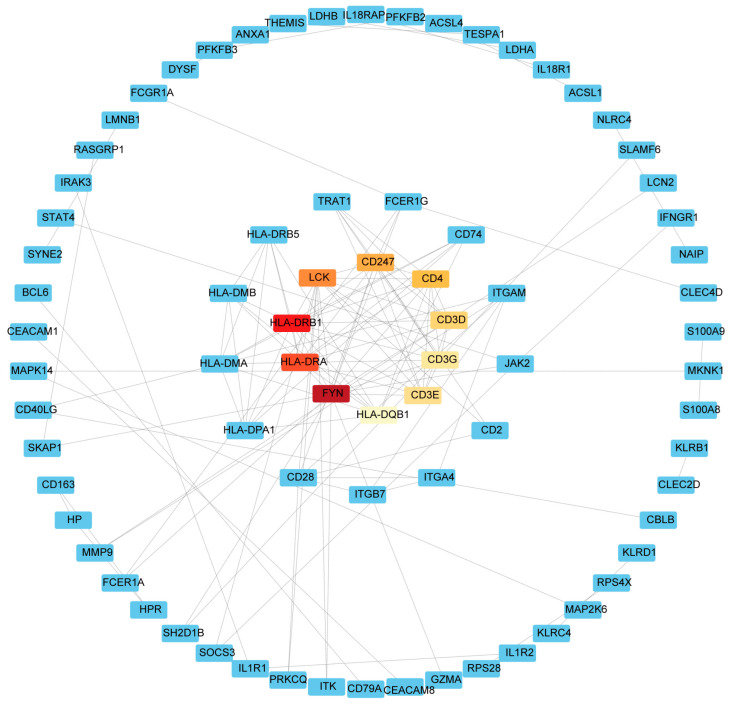
The hub gene in the PPI network was identified using the CytoHubba plug in Cytoscape. Red represents hub genes, darker colors represent hub genes ranked higher.

Common DEGs from COVID-19 and sepsis were uploaded to STRING to explore the network of PPI. PPI network maps were visualized through Cytoscape software to identify common DEG interaction (Figure 4; Supplementary Table S6). Subsequently, we identified the most significant hub genes through degree analytical methods using the CytoHubba plug-in in Cytoscape, including FYN, HLA-DRB1, HLA-DRA, LCK, CD247, CD4, CD3D, CD3E, CD3G, and HLA-DQB1 (Figure 5). Then, we performed the ROC analysis to examine the diagnostic performance of these hub genes for distinguishing disease tissues from normal samples for COVID-19 and sepsis, respectively. Our results showed that all the hub genes had an excellent diagnostic performance in both COVID-19 and sepsis (Supplementary Figures S1 and S2). These data demonstrated, as the highlight of this study, that the hub genes may play an important role in the pathological mechanisms behind COVID-19 and sepsis.

### 2.4. Establishment of TF and miRNA Regulatory Networks

**Figure 6:**
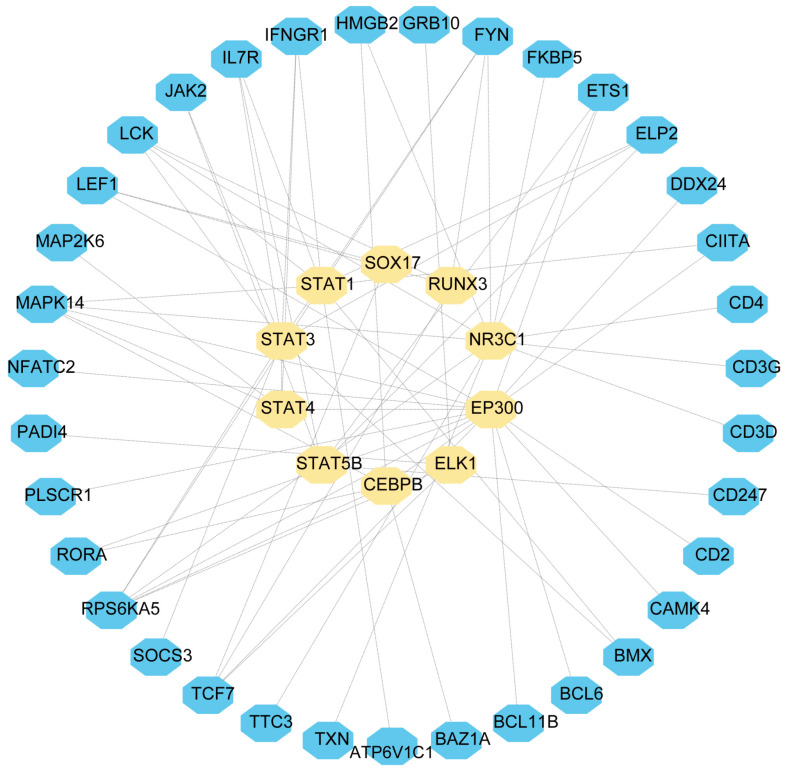
DEGs-TF were used as nodes in an interconnected regulatory network. Light yellow hexagon nodes represent TFs, and genes interact with TFs as blue hexagon nodes.

**Figure 7:**
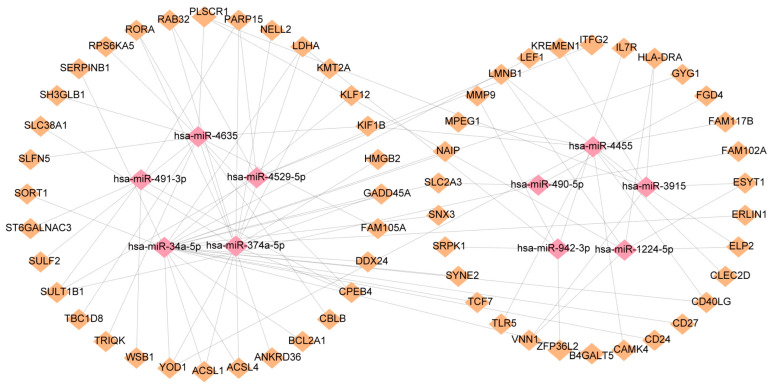
DEGs and miRNAs interact to regulate gene expression. The miRNAs are represented by pink diamond patterns and DEGs are represented by orange diamonds.

In order to elucidate the key molecules that bridge COVID-19 and sepsis, we conducted a framework to indicate gene regulatory networks involving TFs and miRNAs from DEGs, respectively. By analyzing the interaction network of TFs using the EnrichR database, we found 139 potential TFs that regulated common DEGs (Supplementary Table S7). Next, we constructed the interaction network of DEGs with the top 10 TFs according to the combined score (Figure 6). Using the miRTarBase module in EnrichR, we obtained 2095 miRNAs that potentially regulated common DEGs and built the interaction network of DEGs with the top 10 miRNAs (Supplementary Table S8; Figure 7). Our results indicated the potential connections between common DEGs with TFs or miRNA, respectively.

### 2.5. Identifying Potential Drugs

**Figure 8:**
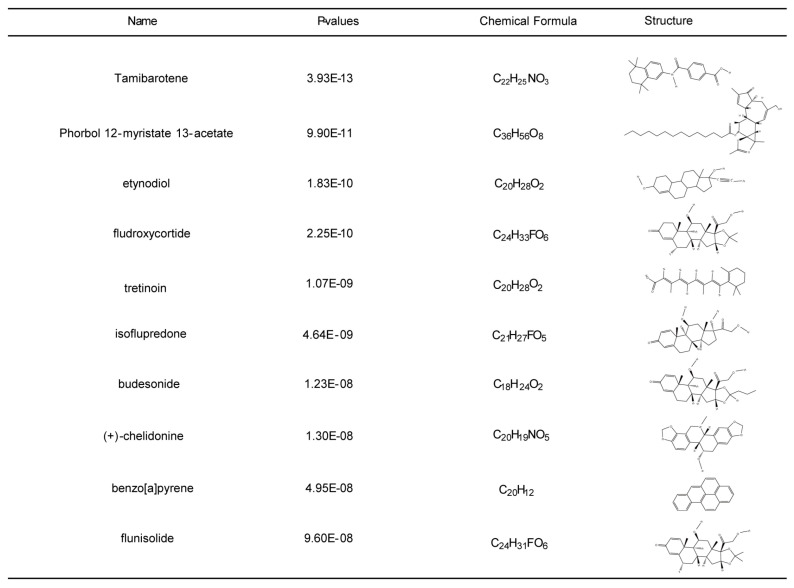
Top ten potential drugs were selected by Enrichr composite score. Ten drug names, *p*-values, molecular formulas, and two-dimensional structures are shown.

In order to shed light on the personalized treatment of COVID-19 and sepsis patients, small molecule medications were located based on the common DEGs utilizing the DSigDB module in the EnrichR database. According to the combined score, the top 10 associated drugs with significant correlations were identified (Figure 8; Supplementary Table S9). These drugs may serve as therapeutic agents for the treatment of COVID-19 and sepsis.

## 3. Discussion

In recent years, an increasing number of studies have demonstrated that there may be potential connections between different diseases. Therefore, interactions between different diseases are a high-potential field that needs to be studied in the future [23,24,25]. In this study, we explored the potential interaction between COVID-19 and sepsis from a unique perspective. The aim of this study is to provide a new direction and potential targets for treating both diseases by unravelling the interaction between sepsis and COVID-19.

Sepsis is a systemic inflammatory response syndrome caused by the invasion of pathogenic microorganisms such as bacteria into the body [10,26]. Progression of sepsis is closely associated with the production of pro- and anti-inflammatory cytokines [12]. Similarly, COVID-19 increases the secretion of proinflammatory cytokines, G-CSF, and chemokines. These factors may excessively activate the innate immunity [27]. Therefore, COVID-19 infection may serve as a high risk factor for the exacerbation of sepsis. In this study, we performed bioinformatics analysis to identify molecular targets that may serve as potential biomarkers and indicated the underlying interaction between sepsis and COVID-19 based on expression profiles from whole blood transcriptional profiles. Extracorporeal blood purification therapy is a very effective method to improve the prognosis of patients with sepsis. Removing the inflammatory mediators or bacterial toxins (or both) from the blood will significantly decrease the host inflammatory response [28]. In addition, a recent study showed that several novel important biomarkers have also been identified in the blood of COVID-19 patients [29,30]. These results demonstrate that blood cells not only participate in the immune system, but also may serve as biomarkers for COVID-19 and sepsis.

After the intersection of differentially expressed genes from COVID-19 and sepsis, we finally obtained 307 common DEGs. For biological processes, the most significantly enriched GO term is T cell activation. Activation of T cells is triggered by intracellular signaling cascades initiated by antigen-activated T cell receptors (TCRs) [31]. Sepsis-induced persistent immune paralysis is defined, in part, by impaired CD4^+^ and CD8αβ^+^ T cell responses in the post-sepsis setting, whereas dysfunction of T cell immunity impacts naive, effector, and memory T cells, and is not restricted to classical CD8αβ^+^ T cells [32]. Sepsis-induced severe and transient lymphopenia is a main factor in decreasing T cell immunity [32]. CD4^+^ and CD8^+^ T cells could be activated by certain antigens in patients with COVID-19 [33]. T cells showed a more stable activation profile in severe COVID-19 patients than in mild patients [34]. For cellular compartment (CC), the most significantly enriched GO term is the external side of plasma membrane. Plasma membrane is important in the viral assembly of SARS-CoV-2 [35]. For molecular function (MF), immune receptor activity and MHC protein complex binding are enriched in GO terms. Loss of platelet MHC-I decreases sepsis-associated mortality in septic mice [36]. In addition, SARS-CoV-2 infection leads to MHC-Ι down-regulation through ORF8 [37].

KEGG is one of the most frequently used databases for functional analysis of genes [22]. In order to explore the most-affected pathways of common DEGs between COVID-19 and sepsis, we performed KEGG enrichment analysis based on the KEGG database. PD-L1 expression and PD-1 checkpoint pathway are significantly enriched in the KEGG pathways. A recent study indicated that severe COVID-19 patients displayed dysregulated expression of checkpoint molecules PD-1 and its ligand PD-L1, indicating that these checkpoint molecules could be considered as prognostic markers and therapeutic targets for COVID-19 [38,39].

PPI networks were constructed using common DEGs to understand the potential biological functional properties of proteins and predict potential biomarkers for COVID-19 and sepsis. Furthermore, we identified 10 hub genes from common DEGs according to the degree algorithm. FYN is crucial for T cell receptor signaling, brain functions, and cell adhesion-mediated signaling [40]. HLA-DRA is an immune-suppressive gene, which may serve as a novel target for immunosuppressive drugs [41]. CD4 is a member of the immunoglobulin superfamily and is mainly expressed in most thymocytes and T cell subsets, and weakly expressed in macrophages and dendritic cells [42]. As a co-receptor of TCR in T cell activation, CD4 also plays a role in thymic differentiation by binding to MHC class II [43]. It has been shown that T cells are regulated by CD4 coreceptor gene expression during development [44]. Moreover, CD4-expressing cells are early mediators of the immune system in septic patients [45]. Furthermore, decreased CD4 expression in lymphocyte subsets has also been observed in COVID-19 patients [46]. CD3D, another hub gene, plays an important role in the transduction of T cell signaling [47]. These hub genes may play an important role in immunotherapy and have great potential as a therapeutic target.

Next, we conducted a framework to elucidate gene regulatory networks involving TFs and miRNAs from common DEGs. It has been reported that dysregulation of MITF may induce severe COVID-19 infections [48]. However, its function in sepsis remains to be addressed. In addition, it has been demonstrated that the miR-491-5p predicting model was detected as a blood-based biomarker for head and neck squamous cell carcinoma in humans [49]. In lung cancer cells treated with apigenin, miR-34a-5p may play an important role in inducing apoptosis through down-regulation of SNAI1 [50]. miR-374a-5p can suppress the proliferation and migration of non-small cell lung cancer cells via targeting NCK1 [51]. These hub genes may have the potential to be promising biomarkers and new targets in therapeutic approaches for COVID-19 and sepsis.

Several drugs have previously been identified as potential COVID-19 therapeutics. Among them, Remdesivir has been approved by the FDA as a promising antiviral drug with a broad-spectrum antiviral activity against RNA viruses, including SARS-CoV, SARS-CoV-2, and hepatitis C virus (HCV) [52,53]. However, several trials have found no statistically significant differences in clinical improvement or mortality between remdesivir-treated and control groups. Therefore, there is still an urgent need to discover novel drugs for the treatment of COVID-19. Ten potential drugs were screened in this study. Fludroxycortide and isoflupredone are used as anti-inflammatory treatment [54,55]. However, their potential value in the treatment of COVID-19 and sepsis need to be determined in the future. Another potential drug, Tamibarotene, has been used in the treatment of COVID-19 [56]. In addition, we are looking for potential drugs that may treat both diseases simultaneously. Budesonide is a synthetic steroid with potent local anti-inflammatory effects and systemic bioavailability, and is likely to be an effective drug for relieving the symptoms of both COVID-19 and sepsis [57,58]. More importantly, Budesonide has also been demonstrated to have therapeutic effects in COVID-19 patients in numerous clinical trials [59,60].

It should be noted that our results still have some limitations. All the above results, including the identification of hub genes, regulatory networks and drug candidates, are based on bioinformatics calculations and analyses. Basic experiments or clinical trials are still needed to verify the biological function of hub genes and the efficacy of drug candidates.

## 4. Materials and Methods

### 4.1. Data Collection

Expression profiles were obtained from the National Center for Biotechnology Information (NCBI) database GEO (https://www.ncbi.nlm.nih.gov/geo/; accessed on 19 August 2022) [61]. In the COVID-19 dataset (GSE179850), RNA-seq profiling was performed on whole blood from 31 COVID-19 patients and 16 healthy donors. Whole blood of healthy control and COVID-19 patients was collected on day 1 (admission day) [62]. The sepsis dataset (GSE134347) contains whole blood RNA-seq data from 156 sepsis patients and 83 healthy individuals [63], and blood was collected within 24 h of ICU admission. Patient characteristics are tabulated in original papers.

### 4.2. COVID-19 and Sepsis Differentially Expressed Genes

Differential analysis was performed using the R software (version 4.0.2) “limma” package and “DESeq2” package. In the GSE179850 dataset, we set the threshold of |log_2_FoldChange| ≥ 0.5 and |adj.P.Val.| < 0.05 to screen reliable differentially expressed genes, while we set the threshold of |log_2_FoldChange| ≥ 1.2 and |adj.P.Val.| < 0.05 to screen reliable differentially expressed genes in the GSE134347 dataset.

### 4.3. GO and KEGG Enrichment Pathway Analysis

Gene Ontology (GO) enrichment analysis and Kyoto Encyclopedia of Genes and Genomes (KEGG) analysis were performed using the “clusterProfiler” package of R software. The q value < 0.05 was used for identifying the significant functional items and pathways.

### 4.4. Analysis of Protein–Protein Interactions (PPIs)

Networks of protein–protein interactions (PPIs) are associated with all kinds of biological processes. Construction of a PPI network will provide insight into molecular processes [64]. Differentially expressed genes were uploaded to STRING (version 11.0) and the median confidence 0.7 was used to construct the PPI network. Cytoscape (v.3.7.2) was used to visualize the PPI network. Both the color and the size of the nodes indicate the score calculated using degree topological analysis methods.

### 4.5. Extraction of Hub Genes

CytoHubba is a plug-in for Cytoscape that allows users to evaluate and identify the key modulators of biological networks based on network metrics [65]. To predict hub genes, we used CytoHubba to screen important nodes in PPI network modules. Determination of the top 10 genes depends on the degree algorithm. The ranks of hub genes are represented by a gradient from red to yellow. Finally, the hub genes were ranked for the shortest accessible paths between hub genes, making them easier to observe.

### 4.6. Identification of Transcription Factors and miRNAs Associated with Common DEGs

Transcription factors (TFs) control chromatin and transcription by identifying specific DNA sequences [66]. In addition to controlling genome expression, they provide essential information for molecular understanding. Enrichr (http://amp.pharm.mssm.edu/Enrichr, accessed on 19 August 2022) is an open-access resource that is a comprehensive gene set enrichment analysis web server [67]. We imported common differential genes to Enrichr to obtain TFs. Subsequently, the top ten TFs were selected according to the composite score and visualized their interaction relationship in Cytoscape. In addition, we also performed gene–miRNA interaction analysis using the miRTarBase module in Enrichr. MiRTarBase is one of the most comprehensive databases of miRNA–target interactions [68]. By exploring gene–miRNA interaction relationships, miRNAs that affect protein expression by disrupting the stability and translation efficiency of target mature mRNA were detected [69]. Similarly, gene–miRNA interaction relationships were visualized in Cytoscape.

### 4.7. Potential Drug Analysis

Analysis of effective potential drugs for COVID-19 and sepsis is one of the purposes of this study. We used the DSigDB module in Enrichr to import common DEGs to detect potentially effective drugs. The DSigDB is an innovative resource for identifying target genes [70]. Subsequently, the top ten potential drugs were selected for further analysis based on the comprehensive ranking. PubChem (https://pubchem.ncbi.nlm.nih.gov, accessed on 19 August 2022) is a repository of information on chemicals and their biological activities for sharing, analyzing, and integrating data from other databases. We used PubChem to download the molecular formulas of potential drugs and their two-dimensional structures to assist drug research.

## 5. Conclusions

In this study, we performed DEGs analysis based on whole blood transcriptome datasets of sepsis and COVID-19. Our study will provide a more reliable therapeutic direction for the treatment of sepsis and COVID-19.

## Data Availability

Any data and R script in this study can be obtained from the corresponding author upon reasonable request. The final manuscript was read and approved by all authors. In this study, publicly available datasets were analyzed. These data are available at NCBI GEO (https://www.ncbi.nlm.nih.gov/).

## Supplementary Materials

The following supporting information can be downloaded at: https://www.mdpi.com/article/10.3390/ijms24032591/s1.
