# Supplementary figures and images for: Peripheral Blood Genes Crosstalk between COVID-19 and Sepsis
Source: Int J Mol Sci. 2023 Jan 30;24(3):2591. doi: 10.3390/ijms24032591 (PMC9916586; doi:10.3390/ijms24032591)

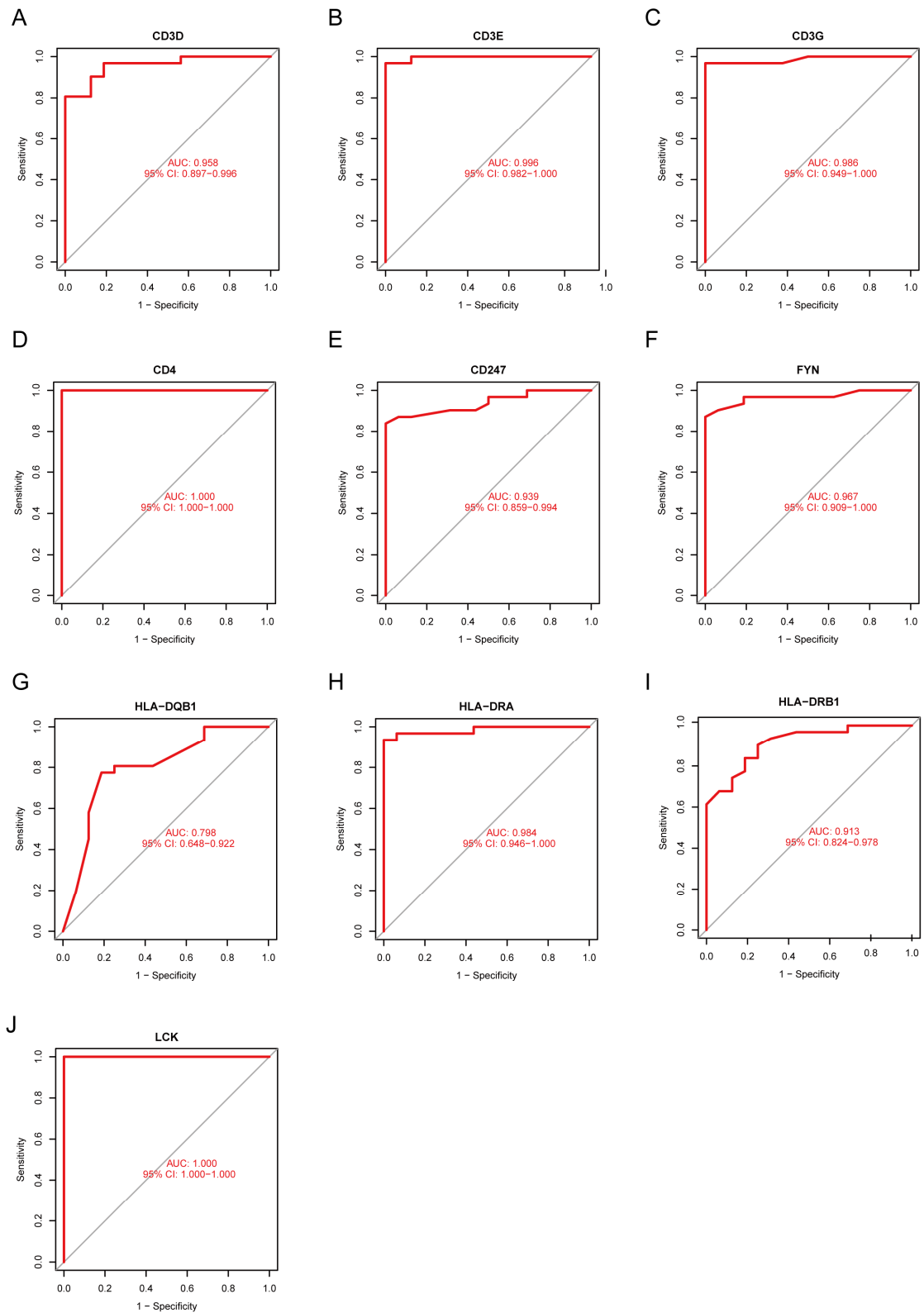

**Supplementary Figure S1:** ROC analysis of the hub DEGs in the COVID-19 dataset.

Supplement: Supplementary file 1 [file ijms-24-02591-s001.zip › Supplementary figure S1.pdf]

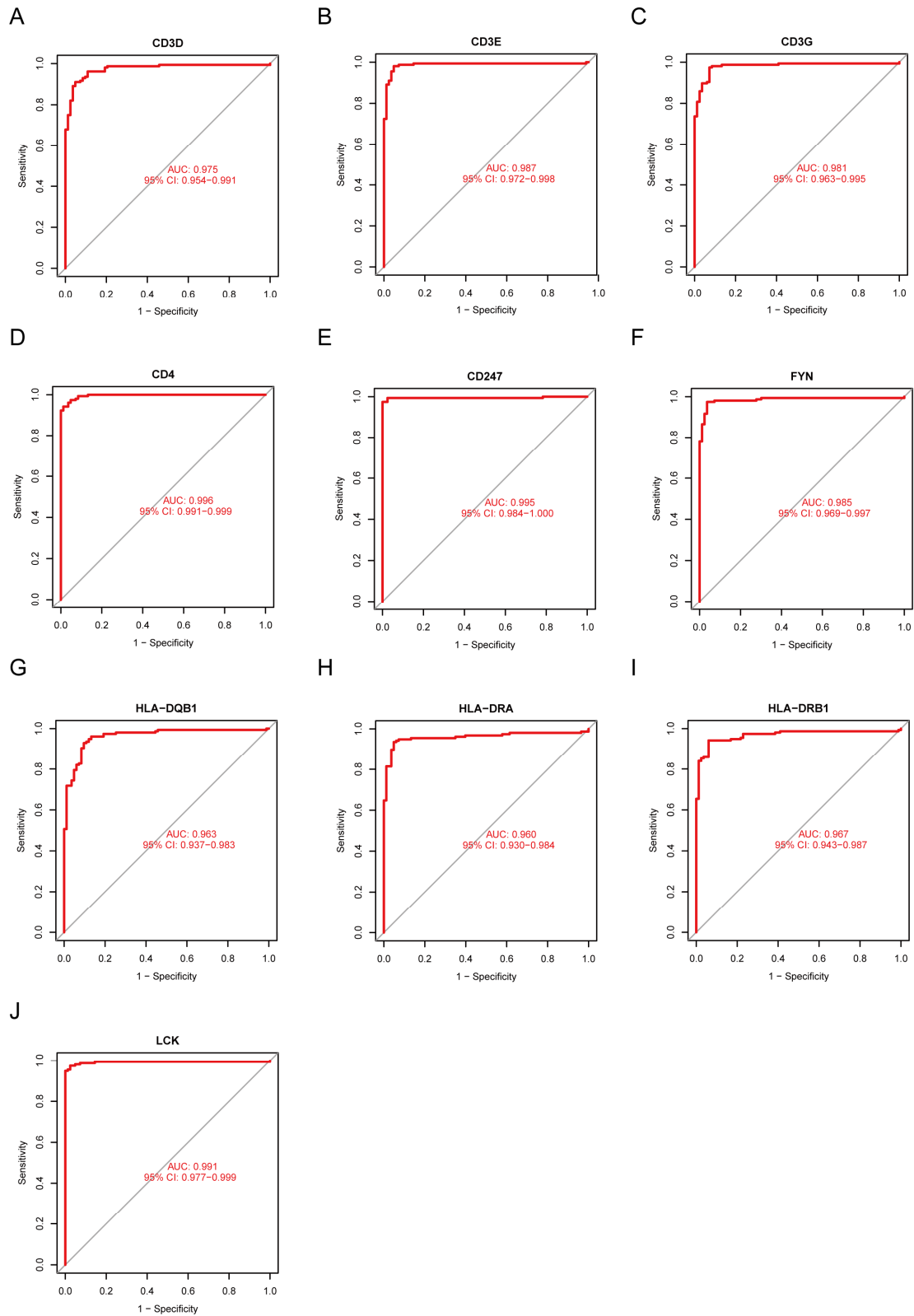

**Supplementary Figure S2:** ROC analysis of the hub DEGs in the sepsis dataset.

Supplement: Supplementary file 1 [file ijms-24-02591-s001.zip › Supplementary figure S2.pdf]
